# Distinct Antiviral Responses in Pluripotent versus Differentiated Cells

**DOI:** 10.1371/journal.ppat.1003865

**Published:** 2014-02-06

**Authors:** Justin M. Pare, Christopher S. Sullivan

**Affiliations:** The University of Texas at Austin, Molecular Biosciences, Austin, Texas, United States of America; Columbia University, United States of America

## Introduction

There is a mystery unfolding, the solution of which has implications for the understanding of both stem cell biology and the evolution of the vertebrate pathogen defense response. At the heart of this puzzle lies the observation of substantially different antiviral responses in mammalian cells with high potency (e.g., embryonic stem, oocytes, induced pluripotent, teratocarcinoma, and embryonic carcinoma cells) versus differentiated somatic cells (i.e., epithelial, fibroblast, lymphocyte). While differentiated cells are proficient in the interferon (IFN)-associated protein-based response [1]–[3], pluripotent cells have an attenuated IFN response [4]–[8]. Conversely, pluripotent cells can utilize RNA interference (RNAi) to combat viruses [9], [10], while this response is attenuated in differentiated cells [11]. Here we provide an overview of this developing area of virology.

## Early Observations of Altered Antiviral Responses in Pluripotent versus Differentiated Cells

Somatic mammalian cells have the capacity to detect double-stranded RNA (dsRNA), a common byproduct of viral replication, and respond by inducing the expression of interferon (reviewed in [12]). Interferon acts in a paracrine and autocrine fashion to induce the expression of hundreds of antiviral interferon-stimulated genes (ISGs), forming the basis of the protein-based antiviral response in mammals [12]. Studies from the 1970s provided the first evidence that pluripotent cells can have altered susceptibility to virus infection. Teratocarcinoma cells were shown to be refractory to infection by murine polyomavirus, while differentiated derivatives of these cells were susceptible [13]. This work inspired further inquiry into infection of undifferentiated cells. Subsequently, Burke et al. demonstrated that pluripotent cells do not produce type I IFN in response to viral infection or treatment with poly I:C, a mimic of double-stranded RNA [4]. In the nearly 40 years since, numerous reports have reiterated these inherent differences [5]–[8]; however, the mechanism is only now being revealed [14]–[16].

## Multiple Components of the Protein-Based Antiviral Response Are Attenuated in Pluripotent Cells

Understanding the basic biology of embryonic stem cells (ESCs) and induced pluripotent stem cells (iPSCs) is an area of intense research. Recent reports establish that these cell types are deficient in numerous components of the IFN and associated protein-based antiviral innate response. Human ESCs display reduced expression of genes involved in the dsRNA response pathways, including pathogen recognition receptors (PRRs) that lead to IFN induction such as OAS1, PKR, MDA5, TLR3, and others [14]. Similar decreases in TLR3 and MDA5 were observed in mouse ESCs [15], demonstrating that this attenuated antiviral response is at least partially conserved among diverse mammals. In addition to reduced dsRNA-mediated induction of IFN, enhanced expression of SOCS1 (an inhibitor of the IFN-activated transcription factor STAT1) contributes to an attenuated response to IFN stimulation in pluripotent cells [16]. Thus, multiple levels of the innate protein-based immune response, both upstream and downstream of IFN production, are attenuated in pluripotent cells.

About 20 years after the reports that the IFN response of embryonic cells is deficient, the discovery of RNA interference (RNAi) led to these observations being revisited. Several studies demonstrated sequence-specific repression of gene expression following the introduction of long dsRNA into *C. elegans*, *Drosophila*, and trypanosomes [17]–[19]. We now know that ∼22 nt, double-stranded RNA duplexes (small interfering RNAs or siRNAs) function as the RNA effectors of RNAi, and that the cytoplasmic exonuclease Dicer can process long dsRNA into mature siRNAs. Argonaute proteins bind the siRNA, forming the RNA-induced silencing complex (RISC), which represses translation and/or directs cleavage of complementary mRNAs. Long dsRNA was effective in eliciting RNAi-mediated silencing in these early experiments, as the invertebrate organisms lack an IFN-based immune response to the presence of dsRNA. However, this approach presented a considerable obstacle for studying RNAi in mammalian cells, which respond to long dsRNA in the cytoplasm by globally inhibiting translation and inducing the interferon response [12]. Because pluripotent cells are deficient in this dsRNA response, several groups were able to overcome this obstacle [5]–[7]. They demonstrated that mammalian cells retained a functional RNAi pathway and affirmed that undifferentiated cells have an attenuated IFN response to long dsRNA.

## RNAi Is Attenuated in Differentiated Cells Undergoing Antiviral Signaling

**Figure 1:**
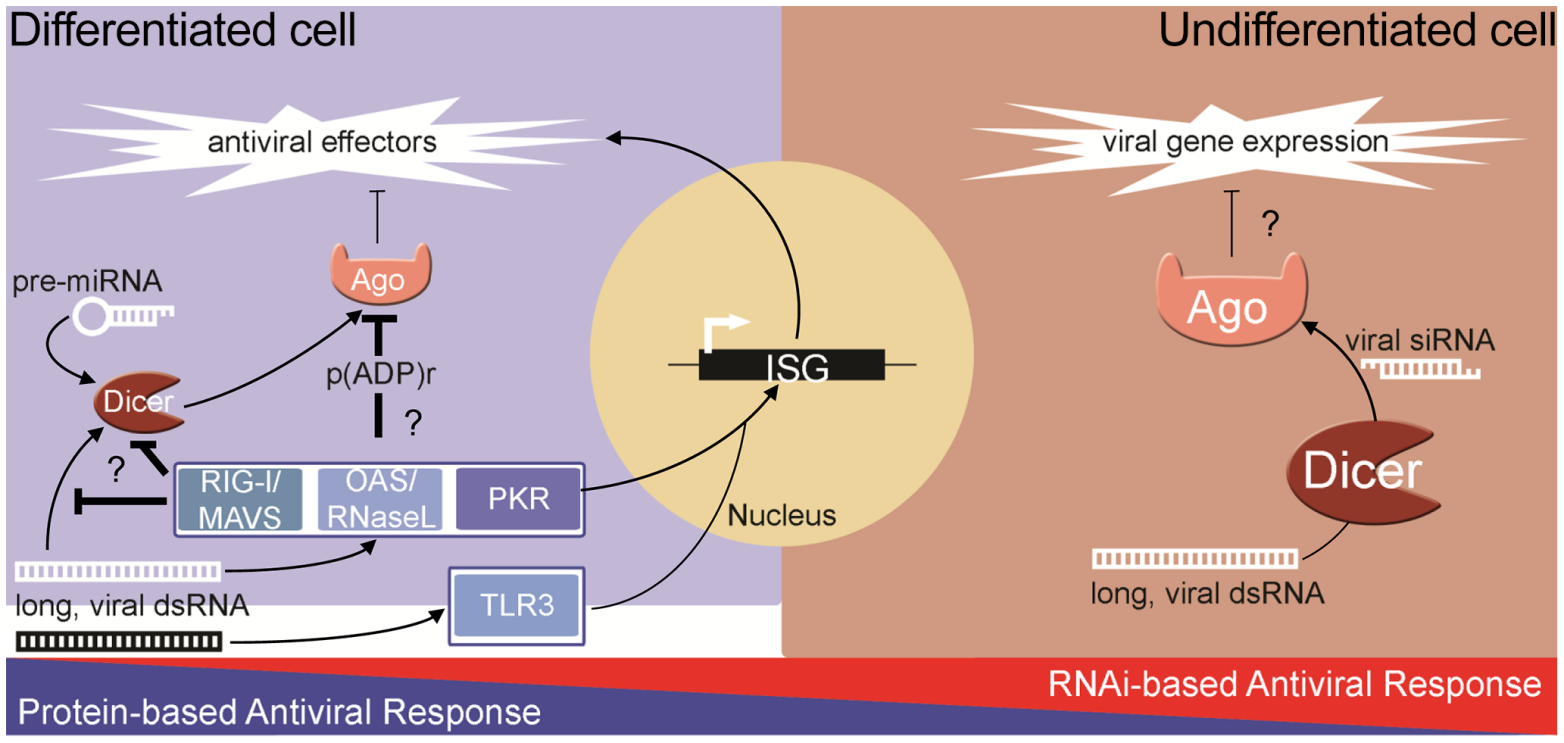
The dichotomy of antiviral responses in differentiated versus undifferentiated cells. In most differentiated cells, PRRs (e.g., RIG-I, OAS-1/RNaseL, PKR) recognize pathogen-associated molecular patterns and stimulate the protein-based interferon response. The RNAi pathway functions, through miRNAs, to temper the expression of cytotoxic transcripts, but is inhibited in response to stress and antiviral signaling pathway activation. In undifferentiated/pluripotent cells, the interferon response is attenuated through reduced expression and activity of numerous components. In these cells, the RNAi pathway can function directly as an antiviral defense, using longer, virally derived dsRNA to generate siRNAs that target and silence viral transcripts.

The ability of the RNAi pathway to combat virus infection is shared among many metazoans including plants and invertebrates. However, it is still debated whether RNAi is an antiviral response in mammals where the complex IFN-based response exists [20]–[23]. Unlike in invertebrates, strong biochemical evidence of natural antiviral siRNAs produced during infection is lacking in differentiated cells. Furthermore, unlike in plants and insects, genetic experiments have failed to demonstrate that the growth of viruses with mutant suppressors of RNAi is rescued in differentiated cells defective for RNAi. Additionally, recent reports from our lab and others have shown that human Argonaute2 (Ago2), a key component of RISC, is inhibited during both stress [24] and the pathogen response [11], [25]. Therefore, if RNAi is to have antiviral function in somatic cells, the coordinated inhibition of Ago2 creates a significant paradox. Thus, converse to the protein-based response to dsRNA in pluripotent cells, at least two components of the RNAi pathway are impaired in differentiated cells: 1) production and/or stability of siRNA; and 2) the activity of Ago2. In contrast to this, recent reports show that RNAi can act as an antiviral response in pluripotent mammalian cells [9], [10]. These findings suggest an intriguing dichotomy whereby differentiated, somatic cells rely on the protein-based IFN response, while undifferentiated cells can utilize RNAi (Figure 1).

## The Advantage of Being Different

MicroRNAs (miRNAs) are small RNAs, derived from endogenous precursors, and, similar to siRNAs, mediate the silencing of mRNAs via RISC. Multiple components of the mammalian pathogen defense response, including some subclasses of ISGs, are regulated by miRNAs. We have proposed that with the evolution of a protein-based antiviral response in most differentiated cells, components of RNAi (no longer essential as protectors against virus) became repurposed to control the toxic effectors of the pathogen defense [11]. Consistent with this, the pathogen response can lead to inhibition of RISC and a subsequent increase in translation of antiviral and/or inflammatory transcripts [11], [25]. This model is in line with the established role of miRNAs as important regulators of homeostasis [26], [27].

## Key Questions Remaining

Despite such recent progress in the field, important questions regarding the different antiviral responses of pluripotent and differentiated cells remain unresolved.

### Pluripotent Cells

What advantage do pluripotent cells gain from lacking multiple arms of the protein-based IFN-associated response? Pluripotent cells undergo rapid cell division, and may mute the IFN response to avoid its antiproliferative effects [28]. Interferon has been shown to stimulate differentiation [28], suggesting that pluripotent cells may inhibit its expression as a means of maintaining potency. An alternative, non–mutually exclusive model predicts that RNAi serves as a more efficient defense against transposons than the IFN response [14]. An extension of this model is that the fitness cost of transposon activity in pluripotent cells is higher than infection by an exogenous virus. Consistent with this proposed role for the RNAi machinery in pluripotent cells, mammalian oocytes have been shown to maintain slicer-dependent RISC activity despite having decreased microRNA-mediated, slicer-independent silencing [29]. Yet another model suggests that relatively “harmless” triggers of the IFN response (i.e., cytoplasmic dsRNA) are readily produced in pluripotent cells and the suppression of the IFN response prevents terminal sacrifice of the lineage. The lack of a protein-based antiviral response in pluripotent cells raises the question of whether the antiviral response is even important in these cells. It is unknown what fraction of pluripotent cells actually come into contact with exogenous virus, and it has even been speculated that such infections would be so detrimental to daughter cells that these cells may purposely not mount a defense to ensure their destruction [14]. Testing these models and understanding the motivation behind attenuating the IFN response gets to the heart of the biology of pluripotency.

### Differentiated Cells

It is unknown what accounts for the lack of abundant detectable siRNAs in somatic cells. Is Dicer or one of its cofactors differentially active and/or are the dsRNA-binding proteins that are components of the IFN response sequestering long dsRNA away from Dicer? Alternatively, are unknown factors altering the stability of the derivative siRNAs or impairing their association with RISC?

Downstream of Dicer, exciting questions remain for understanding antiviral-signaling-mediated inactivation of RISC. For example, what is the full repertoire of PRRs, signaling pathways, effectors, and biochemical changes involved in inhibition of RISC? What are the key infection-relevant miRNAs and associated ISG targets that are altered by RISC inhibition? Finally, how prevalent are these phenomena in other animals? We predict that somatic cells of other vertebrates (positive for IFN response) should also have an attenuated RNAi response when undergoing antiviral signaling. It will be interesting to determine if similar phenomena are observed in invertebrates (IFN negative), which often have separate Agos that specialize in either miRNA-mediated or siRNA-mediated regulation. One exciting possibility is that only miRNA-associated RISCs will be inhibited during cytotoxic stress in these organisms.

## Conclusion

The last few years have shed much light on virus infection of pluripotent cells. It can be expected that this pace of discovery will continue in the near term. This improved grasp of the antiviral response in pluripotent versus differentiated cells will lead to new inroads in RNAi technology, stem cell biology, and understanding the evolution of vertebrates.
